# Effect of an antimicrobial stewardship program in the prevention of antibiotic misuse in patients with spinal cord injury undergoing minor urologic procedures: a single-group, quasi-experiment study

**DOI:** 10.1186/s12879-023-08351-4

**Published:** 2023-06-01

**Authors:** Lina I. Alnajjar, Nourah S. Alrashidi, Njoud Almutairi, Noura Alshamrani, Omar S. Khan, Sajjad Ali, Amira M Youssef, Reem Binsuwaidan

**Affiliations:** 1 Department of Pharmacy Practice, College of Pharmacy, Princess Nourah bint Abdulrahman University, P.O. Box 84428, Riyadh, 11671 Saudi Arabia; 2 College of Pharmacy, Princess Nourah bint Abdulrahman University, P.O. Box 84428, Riyadh, 11671 Saudi Arabia; 3 Infectious Diseases, Medical Affairs Department, Sultan Bin Abdulaziz Humanitarian City, Riyadh, Saudi Arabia; 4 Research and Scientific Center, Sultan Bin Abdulaziz Humanitarian City, Riyadh, Saudi Arabia; 5 Department of Pharmaceutical Sciences, College of Pharmacy, Princess Nourah bint Abdulrahman University, P.O. Box 84428, Riyadh, 11671 Saudi Arabia

**Keywords:** Antimicrobial stewardship program, Minor urological procedure, Spinal cord injury/disease, Antimicrobial prophylaxis, Surgical prophylaxis

## Abstract

**Background:**

Antimicrobial stewardship programs (ASPs) are an internationally recognized strategy for reducing antimicrobial resistance while maintaining patient safety. ASP activities include the restriction of broad-spectrum antibiotics, the establishment of hospital guidelines based on antibiograms, and the promotion of appropriate antibiotic use. This study aimed to determine whether the implementation of antimicrobial stewardship practices improved the effects of a peri-procedure antibiotic prophylaxis prescribed by urologists for patients with spinal cord injury/disease (SCI/D) undergoing minor urological procedures at a tertiary care hospital.

**Methods:**

This single-group, quasi-experiment study included adult patients with SCI/D who required minor urological procedures (cystoscopy, cytobotox, cystolitholapaxy, and urodynamic study) and who were hospitalized between 2012 and 2020.

**Results:**

In total, 233 patients were included in each of the pre- and post-ASP implantation groups. There was a significant reduction in antibiotic use among patients who received a pre-procedure antimicrobial prophylaxis in the post- compared to the pre-implementation group (45.9% vs. 24.46%, *p* < 0.0001), and there was a highly significant reduction in the post- compared to the pre-implementation group in the number who received a post-procedure prophylaxis (16.7% vs. 1.2%, *p* < 0.0001).

**Conclusion:**

ASP implementation is a highly effective strategy for reducing the use of peri-procedure antimicrobial prophylaxes in patients with SCI/D injuries undergoing minor urological procedures.

## Background

Antimicrobial resistance (AMR) has emerged as a worldwide health issue fueled by antibiotic misuse [1]. According to some modeled estimates, by 2050, AMR will lead to the deaths of 2.4 million people globally, with the burden of treatment costing around $3.5 billion annually [2]. Despite these facts and numbers, clinicians often justify antibiotic prophylaxis use because it can prevent multiple post-operative infections. Surgical antibiotic prophylaxis aims to prevent surgical site infections (SSIs) and are thus an essential element of SSI prevention [3]. However, the overuse, misuse, underuse, and abuse of antibiotics lead to AMR [4].

Patients with spinal cord injury/disease (SCI/D) are sensitive to receiving several antibiotic courses. These patients often undergo urological procedures for determining bladder capacity and diagnosing neurogenic bladders. As part of their bladder management program, they require chromic bladder instruments, such as indwelling catheters or intermittent catheterization programs, depending on the bladder management strategy used [5–7].

Recent guidelines for antimicrobial prophylaxis for urological procedures by the American Urological Association (AUA) recommend the use of trimethoprim/sulfamethoxazole (TMP/SMX), the first or second generation of cephalosporins, or aminopenicillins combined with beta-lactamase inhibitors and metronidazole as a single dose less than 24 h before a patient undergoes a minor urological procedure with risk factors including diabetes mellitus, cardiovascular disease, anatomic anomalies, chronic corticosteroid use, and a history of recurrent urinary tract infections (UTIs) [8]. A full course of culture-directed antimicrobial treatment is recommended for documented infection [8]. Guidelines published by the European Association of Urology (EAU) do not recommend the use of antibiotic prophylaxis for minor procedures, including urodynamic study, cystoscopy, and extracorporeal shockwave lithotripsy, to reduce the rate of symptomatic UTIs based on strong rating evidence [9].

Antimicrobial stewardship program (ASPs) are a globally recognized method of reducing antimicrobial resistance while maintaining patient safety. They involve selecting the ideal antimicrobial therapy for each patient, including the dosage and duration, to deliver the best outcomes through optimal therapy [1]. When done correctly, ASPs have led to 22–36% reductions in antimicrobial resistance, and they have also been associated with significant cost reductions in Europe and the United States [10].

Effective antibiotic stewardship guidelines are being implemented in Saudi Arabia to limit inappropriate antibiotic use [10, 11]. With the rising incidence of multi-drug resistant bacterial infections, hospitals must design a program for the judicious use of antimicrobials to control the occurrence of antimicrobial resistance, rationalize antibiotic prescriptions, and eventually improve patient care [12, 13].

The ASP was implemented in 2015 in the studied hospital that is specialized in providing care for patients with SCI/D. An infectious disease consultant, clinical pharmacist, infection control specialist, and clinical microbiologist were part of the ASP team, with activities including reviewing and optimizing the use of prescribed antibiotics, restricting broad-spectrum antibiotics, reviewing angiograms to develop hospital clinical guidelines, and other practices. Thus, the study’s primary objective was to evaluate the appropriateness of using a peri-procedure antibiotic prophylaxis in patients undergoing urological intervention before and after ASP implementation. The secondary objective was to identify gaps in the proper implementation of stewardship. In combination with strong infection-control practices, antibiotic stewardship is likely the best option to combat the looming threat of antibiotic resistance in the absence of a successful line of new antibiotic classes in the near future.

## Methods

### Study design and setting

This single-group, quasi-experiment study was conducted from 2012 to 2020 at Sultan bin Abdulaziz Humanitarian City, a rehabilitation specialist hospital in Riyadh, Saudi Arabia. The hospital provides a wide range of services, including occupational therapy, speech therapy, physiotherapy, social services, rehabilitation services for outpatient clinics, and inpatient admission. Patients with SCI/D comprise a significant number of hospital admissions, and minor urological procedures, such as cystoscopies, were the most common procedures performed.

The ASP was implemented at hospital in 2015. The ASP team implemented hospital guidelines following the EAU, as well as recommended not using antibiotic prophylaxis for minor urological procedures and testing patients undergoing urological procedures for urine culture to allow treatment before the procedure, if necessary. The pre-ASP implementation period was from 2012 to 2015, the post-ASP implementation period was from 2016 to 2020, and guidelines were distributed to all hospital staff.

The ASP interventions the hospital adopted included education (physicians and surgeons), audits, and feedback (real-time and retrospective via phone, email, and reports for the pharmacy and therapeutics committee and infection control committee meetings). Restrictive interventions mostly involved the pre-authorization of restricted antibiotics. However, the implementation of these measures was beyond the scope of this study, so we only assessed guideline concordance according to the number of patients who received a prophylactic antibiotic prescription in terms of its type, dose, and duration. In addition, the timeliness of antibiotic administration pre-surgery was also studied.

### Sampling method, study population, and timeframe

All adult male and female patients who were admitted to the hospital from 2012 to 2020 with SCI/D required a minor urological procedure (cystoscopy, cytobotox, cystolitholapaxy, or urodynamic study). Patients admitted with diagnoses other than SCI/D, pregnant women, patients having undergone pediatric urological procedures, and patients with a current UTI and receiving treatment were excluded. Meanwhile, eligible patients were initially stratified into two groups: one before ASP implementation from 2012 to 2015 (1,787 patients) and a second after ASP implementation from 2016 to 2020 (1,825 patients). We were planning to collect 50% of the sample (250) from the pre-implementation group, with an equal representation of each year (50% / 4 years = 12.5%).

### Data collection

Trained researchers collected data from the health information system (HIS) of the hospital using a predesigned case report form. Data concerning patients’ demographics, including age, gender, comorbidities, medication history by group, voiding methods by assessment, and number of past UTIs were collected. In addition, catheterization-related data, pre- and post-procedure antibiotic use, dose frequency, duration, and other clinical data were collected before and after ASP implementation.

### Study outcomes

The primary objective of the study was to evaluate the appropriateness of prescribing a peri-interventional antibiotic prophylaxis to patients undergoing minor urological procedures pre- and post-ASP implementation. The secondary objective was to assess the gaps in the proper implementation of the ASP.

### Ethical approval and consent to participate

The study was approved in October 2021 via Institutional Review Board (IRB) no# 55-IRB-2021 by the IRB committee in Sultan Bin Abdulaziz Humanitarian City Riyadh, Saudi Arabia (IRB registration number: H-01-R-090), and the need for informed consent was waived. The study was conducted in accordance with the Declaration of Helsinki, and before the analysis, the patients’ information was anonymized and de-identified.

### Statistical analysis

Descriptive statistics included counts and proportions for categorical variables and means and standard deviations (SDs) for continuous variables. The chi-square test (χ2) was used for categorical variables, while the two-tailed student’s t test was used for continuous variables. Antibiotic use practices were compared between pre- and post-ASP implementation based on the surgical prophylaxis guidelines at the hospital, and data analysis was conducted using the Statistical Package for Social Sciences (SPSS) version 21 (SPSS Inc, Armonk, New York, USA), where a p-value of < 0.05 was considered statistically significant.

## Results

**Fig. 1:**
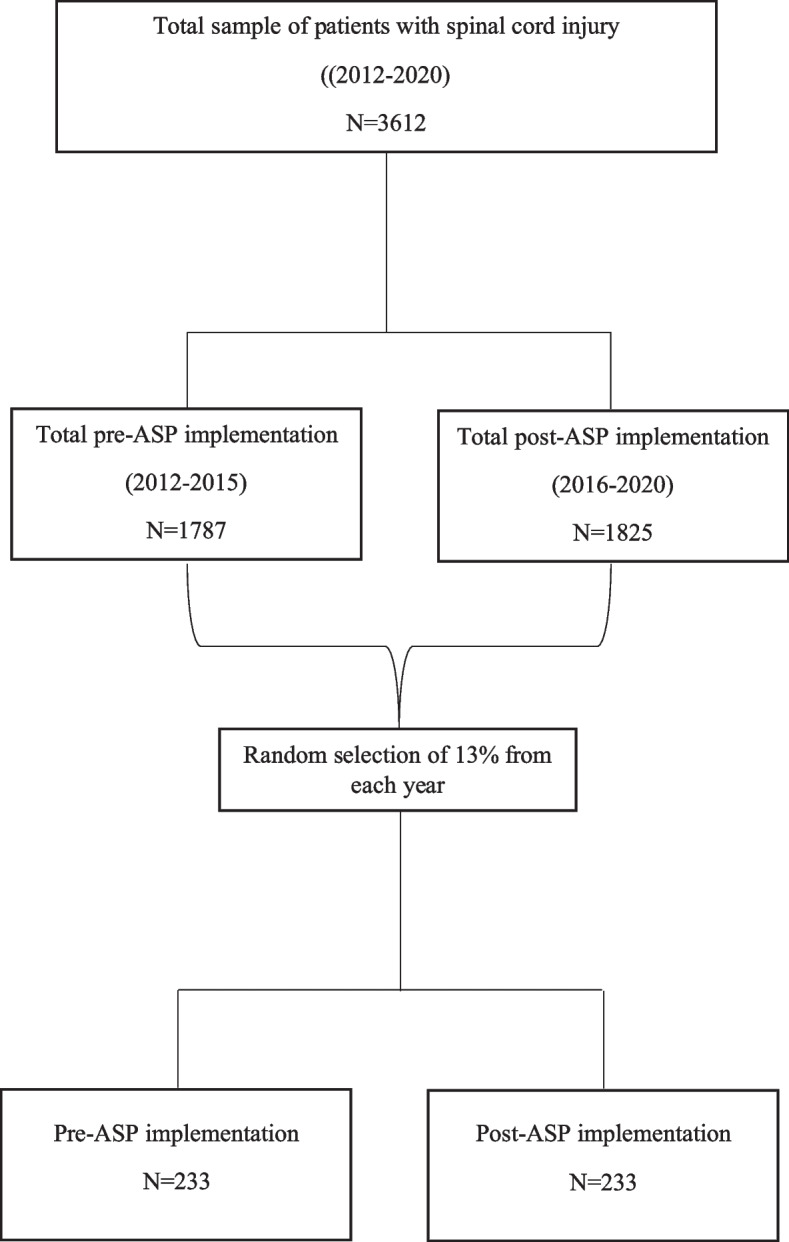
Screening and approach to patient selection

In total, 3,612 eligible adult patients underwent a minor urological procedure from 2012 to 2020: 1,787 patients in the pre-ASP implementation period and 1,825 in the post-ASP implementation period. Around 13% of patients were randomly selected for each year of both the pre- and post-ASP implementation periods, and the total number of patients was 233 patients in each group (Fig. 1).

### Demographic and clinical characteristics

Most of the patients in both groups were male (81.11%), with a mean age of 33 years, and the most common comorbidity observed in these patients was neurogenic bladder, at 91.84% and 99.14% in the pre- and post-ASP implementation groups, respectively. Other comorbidities were significantly fewer in the post-ASP implementation period.

The number of patients with an antimuscarinic drug history was significantly higher in the post- compared to the pre-ASP implementation group (60.51% vs. 41.20%, *P* < 0.0001). Concerning the voiding method, clean intermittent catheterization (CIC) was predominant in both the pre- (51.93%) and post-implementation (54.69%) groups, while the suprapubic voiding method was statistically more predominant in the post- compared to the pre-ASP implementation group (8.15% vs. 2.57%, *P* < 0.007). Further, the number of patients with no previous UTI history was significantly higher in the post- compared to the pre-implementation group (16.73% vs. 6.53%, *P* < 0.0005), while the number of patients who have had at least two past UTIs was significantly greater in the pre- compared to the post-implementation group (41.63% vs. 25.32%, *P* < 0.0002). Further, cystoscopy was the most common minor urological procedure performed in the pre- (33.01%) and post-implementation groups (63.9%), respectively (Table 1).

**Table 1:** Baseline characteristics

	Pre-ASP implementation (*N* = 233)	Post-ASP implementation (*N* = 233)	*p-*value
**Mean age (Y)**	31.65 ± 13.57	34.60 ± 14.73	0.025*
**Gender (n, %)**			
Male	188 (80.86)	190 (81.54)	0.812
Female	45 (19.31)	43 (18.45)	0.812
**Comorbidities (n, %)**			
Neurogenic bladder	214 (91.84)	231 (99.14)	0.0001*
Diabetes	24 (10.30)	27 (11.58)	0.658
Hypertension	11 (4.72)	17 (7.29)	0.243
Dyslipidemia	7 (3)	5 (2.14)	0.557
Other*	46 (19.74)	4 (1.71)	< 0.0001*
**Drug history (n, %)**			
Antimuscarinic	96 (41.20)	141 (60.51)	< 0.0001*
Antibiotics	133 (57.08)	2 (0.85)	< 0.0001*
Alpha-blocker	11 (4.72)	11 (4.72)	1
Other^a^	62 (26.60)	-	-
**Voiding method (n, %)**			
Clean intermittent catheterization	121 (51.93)	124 (54.69)	0.550
Free voiding	99 (42.4)	84 (36.06)	0.1608
Suprapubic	6 (2.57)	19 (8.15)	0.007*
Indwelling	7 (3)	6 (2.57)	0.778
**Number of past urinary tract infections (n, %)**			
0	15 (6.43)	39 (16.73)	0.0005*
1	88 (37.76)	97 (41.63)	0.393
2	97 (41.63)	59 (25.32)	0.0002*
3	12 (5.15)	23 (9.87)	0.053
4	7 (3)	6 (2.57)	0.778
≥ 5	14 (6)	8 (3.43)	0.191
**Surgical procedure (n, %)**			
Cystoscopy	77 (33.04)	149 (63.94)	< 0.0001
Cytobotox	80 (34.33)	45 (19.31)	0.0003
Cystolitholapaxy	14 (6.00)	19 (8.15)	0.3980
Urodynamic study	61 (26.18)	14 (6.00)	< 0.0001
Minor procedure (type not reported)	1 (0.42)	6 (2.57)	0.0561

*Y *Year, N, *n *Number

*Other comorbidities include heart failure, atrial fibrillation, benign prostatic hyperplasia, hyperthyroidism, hypothyroidism, depression, gastroesophageal reflux disease, and peptic ulcer disease

^a^Other drug histories include antidiabetic, antihypertensive, and antidyslipidemic medications

### Antimicrobial prophylaxis pre- and post-ASP implementation

#### Pre-procedure data

**Fig. 2:**
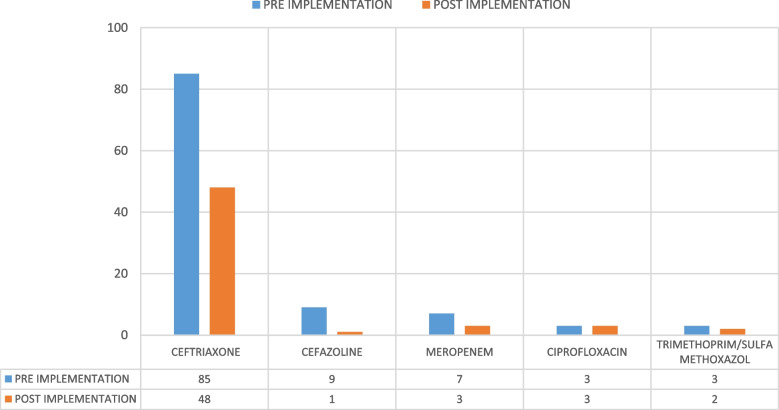
Pre- procedure antibiotic prophylaxis

There was a significant reduction in the number of patients who received an antimicrobial prophylaxis pre-procedure in the post- compared to the pre-implementation group (24.46% vs. 45.9%, *p* < 0.0001), respectively. Further, the number of patients who did not receive an antimicrobial prophylaxis pre-procedure was significantly higher in the post- compared to the pre-implementation group (75.5% vs. 54.08%, *p* < 0.0001). Of the patients who received a surgical antimicrobial prophylaxis, 57 (53.3%) in the pre-implementation group and 55 (96.5%) in the post-implementation group received it within 60 min. Moreover, the number of patients who received an antimicrobial prophylaxis ≥ 60 min was lower in the post-ASP implementation group (3.5% vs. 46.7%, *p* < 0.0001). All patients in both implementation groups received a single-dose antimicrobial prophylaxis (Table 2). In addition, the most administered antimicrobial prophylaxis in both groups was ceftriaxone, in 85 (79.43%) and 48 (84.2%) patients in the pre- and post-implementation groups, respectively (Fig. 2).

**Table 2:** Clinical data of pre-procedure prophylaxis

	Pre-ASP implementation	Post-ASP implementation	*P*-value
**Pre-procedure antibiotic (n, %)**			
Yes	107 (45.92)	57 (24.46)	< 0.0001
No	126 (54.08)	176 (75.54)	< 0.0001
**Timing of surgical antibiotic prophylaxis (n, %)**			
< 60 min	57 (53.3)	55 (96.5)	0.824
≥ 60 min	50 (46.7)	2 (3.5)	< 0.0001
**Antibiotics used (n, %)**			
Ceftriaxone	85 (79.43)	48 (84.2)	< 0.0001
**Frequency (n, %)**			
Once	107 (100)	52 (91.2)	< 0.0001

#### Post-procedure data

There was a highly significant difference between the number of patients in the post- compared to the pre-implementation group who received a post*-*procedure prophylaxis (1.2% vs. 16.7%, *p* < 0.0001), respectively. Patients in both groups received ceftriaxone as a prophylaxis and were given one dose post-procedure. Meanwhile, the number of patients who did not receive a post-procedure prophylaxis was significantly higher in the post- compared to the pre-implementation group (98.7% vs. 83.26%, *p* < 0.0001; Table 3).

**Table 3:** Clinical data of post-procedure prophylaxis

	Pre-ASP implementation	Post-ASP implementation	*P*-value
**Post-procedure antibiotic (n, %)**			
Yes	39 (16.73)	3 (1.28)	< 0.0001
No	194 (83.26)	230 (98.7)	< 0.0001
**Frequency (n, %)**			
Once	39 (100)	3 (100)	-

#### Assessment of the ASP implementation guideline

The results show that surgeons commonly prescribe antibiotics pre-procedure, at a rate of 24.46% in the post-ASP implantation group, and the most common antibiotic was ceftriaxone, given to 84.2% of patients.

## Discussion

Objectively measuring the impact of an ASP on antibiotic use in a healthcare facility is an essential component of any ASP strategy to assess its benefits and pitfalls [2]. Patients with SCI/D pose a challenge in terms of antimicrobial resistance, as they have a unique set of disease parameters depending on the bladder care program in which they are partaking [14, 15]. Between both study groups, CIC was the predominant method of voiding in the pre- and post-implementation groups. This, among other variables, often correlates with the risk of harboring resistant microorganisms and manifesting UTIs. Meanwhile, the high prevalence of asymptomatic bacteriuria thus renders the judicious use of antibiotics, especially as a surgical prophylaxis in these patients, of paramount importance to protect against developing resistance [14].

Inappropriate prescribing of antibiotics was reported as one of the challenges before implementation of the ASP at the studied hospital. This provided a cynosure through which antibiotic prophylaxis misuse could be studied and tested. The results show improvements in prescribing practices after ASP implementation in terms of both pre- and post-procedure antimicrobial prophylaxis.

First, there was a marked reduction in the number of patients who were prescribed antibiotics pre-procedure, which is in accordance with the EUA recommendation against using routine prophylaxis. In terms of post-procedure prophylaxis, similar trends in practice improvements toward not prescribing antibiotic prophylaxis were observed. The duration of an antibiotic prophylaxis was appropriate in both groups (one day, or a single dose on call to the operating room).

Another factor was the timeline of the antibiotic’s prophylaxis administration within the cut-off of 60 min, in accordance with the Infectious Disease Society of America (IDSA) surgical prophylaxis guideline (Bratzler et al., 2013). A marked difference was observed between both groups regarding the timeliness of antibiotic administration, with only two patients out of 57 (3.5%) crossing the 60-min cut-off in the post-implementation period. Ceftriaxone, third-generation cephalosporin, was still preferred over the recommended cefazolin as an intravenous agent, and concessions were made in the hospital by the stewardship committee on account of the local antibiogram. However, there was a significant shift toward not prescribing surgical prophylaxis in the post-ASP implementation period. Improvements in antibiotic consumption, that is, toward using narrow-spectrum antibiotics, must be addressed, with emphasis on not using antibiotics in low-risk patients.

Ceftriaxone, whose high consumption supports the results of previous studies on using broader-spectrum antibiotics in Saudi Arabia as surgical prophylaxis (Balkhy et al., 2018), was also used in low-risk groups. We noted a lack of surveillance of the resistance patterns of prevalent strains and a reduction in antibiotic consumption in the policy dictating the prescription of these agents. In addition, surgeons preferred using third-generation cephalosporins as a routine practice, with concessions granted based on constant consideration of the risk factors involved with SCI/D patients among high-risk patients. Another factor involved was the limited hospital formulary and routine screening of urine cultures, which reported ceftriaxone sensitivity.

The results demonstrate a gap in the adherence of surgeons to the implemented guideline of not prescribing peri-operative antibiotics to patients undergoing minor urological procedures. Continuous feedback and auditing, education, and quarterly reporting must be adopted to improve adherence to the implemented guideline and to reduce antibiotic misuse.

### Limitations

Because of the limitation of our quasi-experiment study design, the findings of similar local studies on antimicrobial consumption are incomparable to our data, as they were based more on prospective ASP interventions. However, the findings of our observations support previous reports that an effective ASP improves antibiotic use and, in turn, improves patient outcomes (File et al., 2014). Some of the other limitations include our inability to identify which components of the ASP were the most effective at reducing peri-operative antibiotic use, as we did not study the interventions prospectively. Third, we did not cover any other condition together with neurogenic bladder, such as stroke or non-traumatic SCI, and the selection of patients for each year at random was a limitation, as we did not cover the total number of patients screened. Lastly, comorbidities were not studied to determine whether surgeons were prescribing peri-operative antibiotics to high-risk patients.

## Conclusion

An ASP for SCI/D patients in a rehabilitation hospital is an example of a highly needed setting, and utilizing related data will greatly improve the appropriate use of antimicrobial therapy, as well as promote prescribers’ acceptance of the guideline, thus reducing related costs and antimicrobial consumption. Future studies should examine the generalizability of these findings to other patient population groups with similar conditions as neurogenic bladder (patients with stroke and non-traumatic SCI/D). Moreover, more studies are needed to assess the long-term clinical benefits for patients of all diseases (mortality benefits, symptomatic UTIs with resistant organisms, recurrent hospitalizations). These studies can ensure the allocation of further resources that are crucial to supporting the expansion of the ASP as it becomes increasingly accepted as a standard of care in many advanced hospital settings.

## Data Availability

The datasets generated and/or analyzed during the current study are not publicly available due to privacy/ethical restrictions, but are available from the corresponding author on reasonable request.

## Abbreviations

ASP: Antimicrobial stewardship program
AMR: Antimicrobial resistance
SSI: Surgical site infection
SCI/D: spinal cord injury /diseases
HAI: Hospital-acquired infection
TMP-SMX: Trimethoprim/sulfamethoxazole
IRB: Institutional Review Board
UTI: Urinary tract infection
ASIA: American Spinal Injury Association
AUA: American Urological Association
EAU: European Association of Urology
IDSA: Infectious Disease Society of America
